# Ankrd2 may alleviate denervation-induced skeletal muscle atrophy by inhibiting inflammation

**DOI:** 10.55730/1300-0152.2793

**Published:** 2025-12-30

**Authors:** Yalin ZHANG, Lin ZHANG, Li LI, Liqin ZHANG, Li ZENG, Jiaying QIU, Zhengrong XI, Wenwen WANG

**Affiliations:** 1 Department of Gynecology, Affiliated Maternity and Child Health Care Hospital of Nantong University, Nantong, Jiangsu, China; 2 Department of Pharmacy, Affiliated Maternity and Child Health Care Hospital of Nantong University, Nantong, Jiangsu, China; 3 Medical School of Nantong University, Nantong, Jiangsu, China; 4 Department of Mass Health Care, Affiliated Maternity and Child Health Care Hospital of Nantong University, Nantong, Jiangsu, China

**Keywords:** Ankrd2, denervation, skeletal muscle atrophy, inflammation, NF-κB signaling pathway

## Abstract

**Background/aim:**

Sciatic nerve injury causes a loss of skeletal muscle innervation, reduced motor function, and eventual muscle atrophy. Inflammation and increased protein degradation are key factors contributing to muscle atrophy. Inflammation is activated early during muscle atrophy and can be modulated by various factors. However, the precise role of inflammation in denervation-induced muscle atrophy remains unclear.

**Materials and methods:**

Transcriptome sequencing was used to determine that the inflammatory response occurs early during denervation-induced muscle atrophy. RT-qPCR validation of several inflammatory factors showed rapid upregulation at early stages, followed by gradual downregulation. Weighted gene coexpression network analysis of differentially expressed genes identified gene modules whose expression patterns were correlated with or inversely correlated to the inflammatory phenotype, thereby identifying key regulatory factors. A total of 14 coexpression modules were identified, and expression patterns opposite to those of inflammatory factors were examined to investigate potential regulatory molecules that could inhibit inflammation and protect skeletal muscle.

**Results:**

Ankrd2 was identified in the darkorange module, showing no significant change at 36 h postdenervation, followed by gradual upregulation, which was opposite to the expression of inflammatory factors. An Ankrd2-overexpressing lentivirus was injected into the tibialis anterior muscle, and Ankrd2 overexpression was found to significantly alleviate muscle atrophy. Gene ontology and Kyoto Encyclopedia of Genes and Genomes analyses showed that Ankrd2 overexpression was associated with downregulation of inflammation-related pathways, particularly the NF-κB signaling pathway. Proatrophy genes in both the ubiquitin-proteasome and autophagic-lysosomal systems were also suppressed.

**Conclusion:**

The present study suggests that denervation-induced muscle atrophy is alleviated by Ankrd2, potentially through inhibition of inflammation, highlighting its potential as a therapeutic target.

## 1. Introduction

Skeletal muscle atrophy, characterized by a significant loss of muscle mass and function, commonly occurs as a consequence of nerve injury. Neuromuscular signaling is disrupted by denervation, leading to progressive deterioration of muscle cell physiology. This process is marked by a reduction in muscle fiber diameter, decreased cell volume, and increased fibrosis, collectively contributing to functional decline (Karam et al., 2017; Zuccaro et al., 2023). At the molecular level, denervation shifts the balance between protein synthesis and degradation, accelerating the breakdown of muscle proteins and driving atrophy. Inflammation is a critical mediator in denervation-induced muscle atrophy. Following nerve injury, immune cells such as macrophages infiltrate muscle tissue and release proinflammatory cytokines, including tumor necrosis factor-α (TNF-α) and interleukin-6 (IL-6) (Ji et al., 2022). These cytokines activate key signaling pathways that promote proteolysis and muscle wasting (Lynch et al., 2007; Wang et al., 2021; Chen et al., 2023). Despite progress in understanding inflammatory mechanisms, the full spectrum of molecular regulators, particularly those that may counteract muscle atrophy, remains incompletely defined.

Transcriptomic approaches such as RNA sequencing have enabled systematic profiling of gene expression changes during muscle atrophy. Previous studies have revealed widespread alterations in transcription and alternative splicing in denervated muscle (Qiu et al., 2021). However, many of these studies have not fully identified key regulatory genes or their functional roles. Weighted gene coexpression network analysis (WGCNA) offers a powerful tool to address this gap by grouping genes into modules based on expression patterns and correlating them with phenotypic traits. For instance, WGCNA has been used to identify atrophy-related genes, such as CUL3 and COPS5, which are involved in ubiquitin-mediated proteolysis in sarcopenia (Xu et al., 2022), as well as the CEBPA–FGF21 network in diabetic muscle atrophy (Wu et al., 2023).

Ankrd2, a member of the muscle ankyrin repeat protein (MARP) family, is highly expressed in skeletal muscle and participates in mechanotransduction and cellular stress responses (Mohamed et al., 2010). Its expression increases in response to muscle stretch and injury, and it has been implicated in the regulation of apoptosis and muscle differentiation through pathways such as PI3K/Akt and MAPK (Bean et al., 2008; Cenni et al., 2011). Elevated Ankrd2 levels in conditions such as muscular dystrophy and cancer cachexia also suggest a potential role as a biomarker or therapeutic target (Kojic et al., 2004). Nevertheless, the functional contribution of Ankrd2 to denervation-induced atrophy—especially its relationship with inflammatory signaling—has not been thoroughly investigated.

In the present study, transcriptome sequencing of skeletal muscle was performed at various time points after nerve disarticulation to analyze gene expression changes, and activation of the inflammatory response was confirmed at 36 h postinjury. WGCNA was used to analyze expression modules correlated with the inflammatory response, and key genes were identified. Ankrd2 was identified in the darkorange module, showing an expression trend opposite to that of inflammatory factors. Ankrd2 overexpression lentivirus was injected into the tibialis anterior (TA), and skeletal muscle atrophy was significantly alleviated, accompanied by inhibition of the NF-κB signaling pathway and reduced expression of proatrophy genes. The present study suggests that denervated muscle atrophy is mitigated by Ankrd2, potentially by inhibiting inflammation, thereby enhancing understanding of its regulatory mechanisms and supporting Ankrd2 as a potential therapeutic target.

## 2. Materials and methods

### 2.1. Animals and experimental design

All experiments involving mice were conducted under a project license from the Animal Ethics Board of Nantong University, in accordance with national guidelines for animal care and use. Adult male C57BL/6 mice (25 ± 2 g) were obtained from the Laboratory Animal Center of Nantong University, (Nantong, China). The mice were housed in a specific pathogen-free facility at 23 ± 2 °C, 60% humidity, with a 12 h light/dark cycle and free access to standard chow and water. A total of 24 mice were randomly assigned to four groups. The control group consisted of six randomly selected mice, in which the sciatic nerve was exposed but not transected. The remaining 18 mice underwent sciatic nerve transection. Samples were collected at 0 h, 36 h, 3 days, and 7 days for RNA-sequencing and morphological analysis. For RNA-sequencing, at 0 h, 36 h, 3 days, and 7 days, the total RNA from three animals was pooled into one sample per time point (n = 1 pooled sample per time point, each pool from three animals). For morphological analysis, samples were collected from three independent animals per time point (n = 3 biological replicates per time point). To ensure sufficient RNA for sequencing, samples from three mice were pooled at each time point. For transfection, the operated mice were randomly divided into groups to receive TA muscle injections starting on the day of surgery. The experimental groups were defined as follows: Normal: sham-operated, nondenervated group; Control: denervated group injected with the corresponding negative control lentivirus (LV-NC); Ankrd2-OE: denervated group injected with LV-Ankrd2; Negative control (NC): denervated group injected with the corresponding negative control lentivirus (shNC); and shAnkrd2: denervated group injected with shAnkrd2. Samples for morphological analysis were collected from each group (n = 4). TA muscles were harvested on the 10th day postsurgery.

### 2.2. Lentivirus construction and intramuscular injection

The full-length coding sequence of mouse Ankrd2 was cloned into the GL214 vector to generate the overexpression construct (LV-Ankrd2). A nontargeting scrambled sequence was cloned into the same vector to serve as the negative control (LV-NC). For Ankrd2 inhibition, a short hairpin RNA (shRNA) targeting mouse Ankrd2 (shAnkrd2) and a nontargeting control shRNA (shNC) were designed and inserted into the pLKO.1 vector. All constructs were verified by DNA sequencing. Lentiviruses were packaged in HEK293T cells using the psPAX2 and pMD2.G packaging plasmids. The viral supernatant was collected at 48 and 72 h posttransfection, concentrated by ultracentrifugation, and titrated to 1 × 10^8^ TU/mL. On the day of sciatic nerve transection surgery, mice in the transfection groups received intramuscular injections into the tibialis anterior (TA) muscle. A total volume of 20 μL containing 1 × 10^6^ TU of either LV-Ankrd2, LV-NC, shAnkrd2, or shNC was slowly injected at three different sites along the length of the TA muscle using a 30-gauge Hamilton syringe (Hamilton Company, Reno, NV, USA).

### 2.3. RNA extraction and quantification

Total RNA was extracted using TRIzol reagent (R411-01; Vazyme, Nanjing, China) and reverse-transcribed into cDNA using the PrimeScript RT reagent kit (R323-01; Vazyme, Nanjing, China). RT-qPCR was performed using SYBR Green (Q111-02; Vazyme, Nanjing, China). The gene primer sequences are listed in Table. Gene expression levels were quantified using the 2^−ΔΔCt^ method and normalized to GAPDH.

### 2.4. Western blot analysis

Total protein was extracted from skeletal muscle using RIPA lysis buffer (P0013B; Beyotime, Shanghai, China) supplemented with protease and phosphatase inhibitors. The protein concentration was measured using the BCA assay kit (P0028; Beyotime, Shanghai, China). A total of 30 μg of protein per sample was loaded and separated on precast 10% SDS–PAGE gels (PG112; Epizyme Biomedical Technology, Shanghai, China). The separated proteins were then transferred to polyvinylidene fluoride membranes (IPVH00010; Millipore, Burlington, MA, USA). Membranes were blocked with 5% skim milk at room temperature for 2 h. Following blocking, the membranes were incubated with primary antibodies diluted in the same blocking buffer overnight at 4 °C. The primary antibodies used were rabbit anti-ANKRD2 (1:1000, 11821-1-AP; Proteintech, Wuhan, China), rabbit anti-CSRP3 (1:1000, ab173301; Abcam, Cambridge, MA, USA), rabbit anti-ATG7 (1:1000, 10088-2-AP; Proteintech, Wuhan, China), rabbit anti-PINK1 (1:1000, 23274-1-AP; Proteintech, Wuhan, China), mouse anti-BNIP3 (1:1000, 68091-1-Ig; Proteintech, Wuhan, China), and rabbit anti-β-tubulin (1:1000, 10068-1-AP; Proteintech, Wuhan, China); which was used as the loading control. The next day, membranes were washed three times (10 min each) with TBST and then incubated with corresponding HRP-conjugated secondary antibodies—goat antimouse (1:2000, SA00001-1; Proteintech, Wuhan, China) or goat antirabbit (1:2000, SA00001-2; Proteintech, Wuhan, China)—for 2 h at room temperature. After incubation, membranes were washed again three times (10 min each) with TBST. Protein bands were detected using ECL (32109; Thermo Fisher Scientific, Waltham, MA, USA) on the Tanon 4200 imaging system (Tanon Science and Technology Co. Ltd., Shanghai, China). Multiple exposure times (ranging from 10 s to 5 min) were tested for each membrane to ensure optimal signal without saturation. Quantification was performed using Image Lab software (Bio-Rad Laboratories, Hercules, CA, USA).

### 2.5. Immunofluorescence staining

Tibialis anterior muscle tissues were fixed in 4% paraformaldehyde, gradually dehydrated in sucrose solutions (10%, 20%, and 30%), and embedded in optimal cutting temperature (O.C.T.) compound. The samples were sectioned into 10 μm-thick slices using a cryostat. The tissue sections were permeabilized with 0.5% Triton X-100 (Sigma-Aldrich, St. Louis, MO, USA) in phosphate-buffered saline (PBS) for 20 min and then blocked with 5% bovine serum albumin in PBS for 1 h at room temperature to reduce nonspecific binding. The tissue sections were incubated overnight at 4 °C with rabbit antilaminin antibody (1:200, ab11575; Abcam, Cambridge, MA, USA). The sections were then incubated with Alexa Fluor 488–conjugated goat antirabbit secondary antibody (1:1000, ab150077; Abcam, Cambridge, MA, USA) at room temperature for 2 h. After washing, nuclei were stained with DAPI (1 μg/Ml, C1006; Beyotime, Shanghai, China) for 5 min. Images were acquired using a confocal laser scanning microscope (Carl Zeiss AG, Oberkochen, Germany), and five fields per sample were analyzed.

### 2.6. Identification of differentially expressed genes

To identify differentially expressed genes (DEGs), DESeq software was used to normalize read counts for each gene and calculate fold changes, followed by a negative binomial distribution test to determine the p value for differences in read counts. In this study, DEGs were considered significantly different if p < 0.05 and the fold change was >2. Venn diagrams were generated using the free online analysis tool OmicShare (Gene Denovo Biotechnology Co. Ltd., Guangzhou, China).

### 2.7. Weighted gene coexpression network analysis (WGCNA)

WGCNA was performed using the R package to analyze the coexpression patterns of DEGs related to muscle atrophy in mice. The soft-threshold power (β) was calculated using the scale-free topology criterion to generate a weighted adjacency matrix. Gene modules were then identified using the dynamic tree cut method. The correlation between each gene module and sample phenotypes was analyzed using the WGCNA package, and the module most strongly correlated with time was selected as the target module.

### 2.8. Functional analysis

The OmicShare tool was used to analyze gene set functions, including biological process (BP), cellular component (CC), molecular function (MF), and Kyoto Encyclopedia of Genes and Genomes (KEGG) pathway analyses to identify pathways associated with the DEGs. Gene ontology (GO) analysis was performed to examine downregulated genes. The p value for each pathway was used to determine the significance of the association between the gene set and the pathway.

### 2.9. Statistical analysis

Statistical analyses were conducted using GraphPad Prism 7 software (GraphPad Software, San Diego, CA, USA), and data are presented as the mean ± standard deviation (mean ± SD). For comparisons between two groups, an unpaired student’s t-test was used. For comparisons involving multiple groups, one-way analysis of variance (ANOVA) was used, and intergroup differences were assessed using Tukey’s multiple comparisons test. A p < 0.05 was considered statistically significant.

## 3. Results

### 3.1. Identification of differentially expressed genes

**Figure 1:**
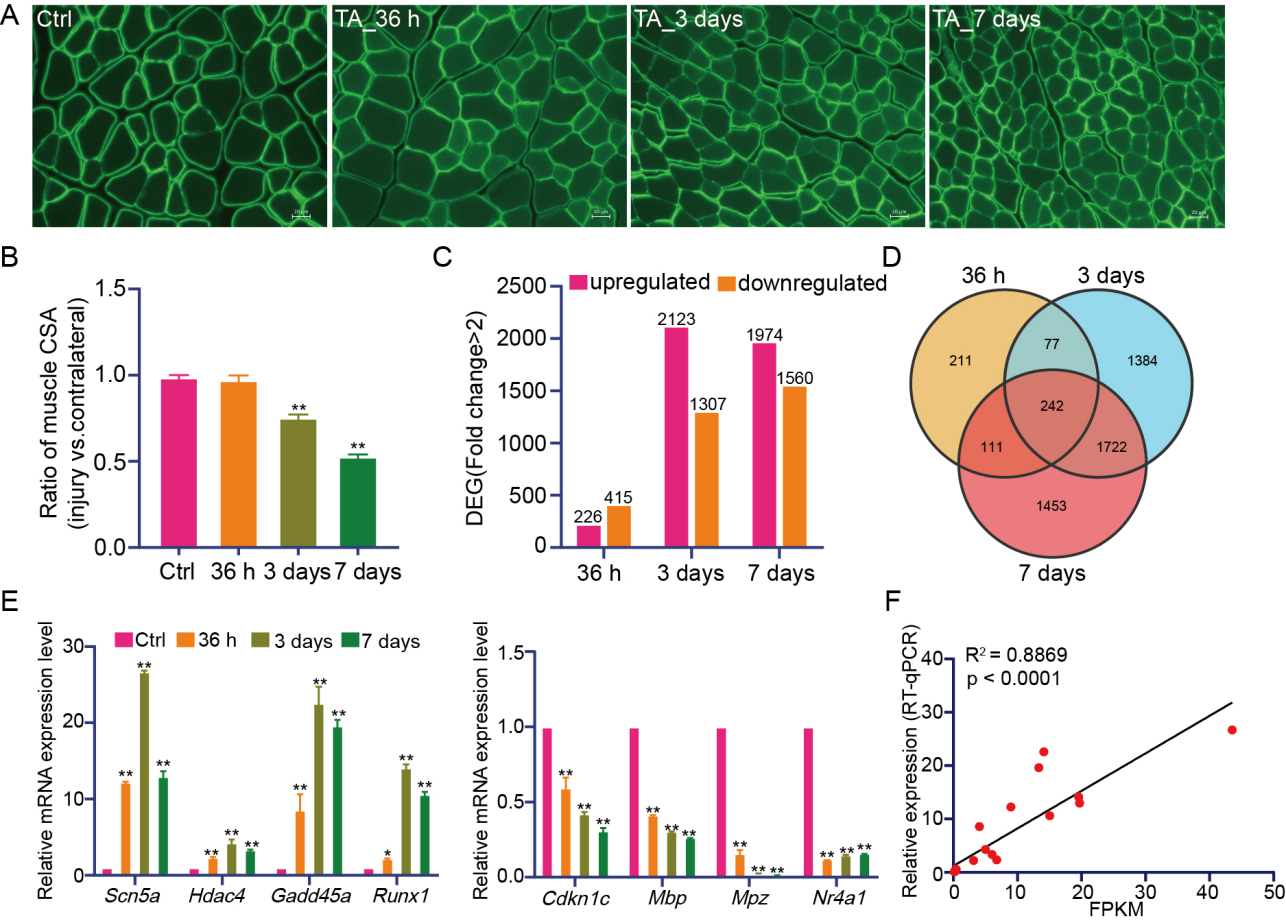
Identification of differentially expressed genes. (A) Immunofluorescence staining of laminin in the tibialis anterior muscle at different time points. Scale bar = 20 μm. (B) Cross-sectional area (CSA) of tibialis anterior muscle fibers. N = 3 mice per group; **p < 0.01 versus the Ctrl group. (C) Summary of the number of DEGs in the tibialis anterior muscle after denervation. Pink indicates upregulated genes, and orange indicates downregulated genes. (D) Venn diagram showing the overlap of DEGs in the tibialis anterior muscle after denervation. (E) Relative expression levels of eight selected upregulated and downregulated genes were calculated relative to *Gapdh* mRNA levels. N = 3 mice per group; *p < 0.05, **p < 0.01 versus the Ctrl group. (F) Correlation between RT-qPCR and RNA-seq data; the correlation coefficient (R) was 0.8869. FPKM, fragments per kilobase of exon per million mapped reads; RT-qPCR, reverse transcription quantitative polymerase chain reaction.

Morphological analysis of the TA muscle from each experimental group was performed using laminin staining to visualize myofiber morphology (Figure 1A). Analysis of myofiber cross-sectional area revealed no significant changes at 36 h, whereas significant reductions were observed at 3 and 7 days compared with the control group (Figure 1B). Bioinformatics analysis (p < 0.05; FC > 2) identified 226 upregulated and 415 downregulated genes at 36 h, 2123 upregulated and 1307 downregulated genes at 3 days, and 1974 upregulated and 1560 downregulated genes at 7 days compared with the control group (Figure 1C). Venn diagrams were generated to analyze common differentially expressed genes among the groups. The results indicated that 242 genes were commonly differentially expressed across the three groups (Figure 1D). Several genes were selected for validation, and the RT-qPCR results are presented in Figure 1E. Correlation analysis between RNA sequencing and RT-qPCR results revealed a correlation coefficient of 0.8869, indicating a strong positive correlation (Figure 1F). These findings demonstrate that the RNA sequencing data are accurate and reliable for subsequent analyses.

### 3.2. Inflammation is activated at an early stage

**Figure 2:**
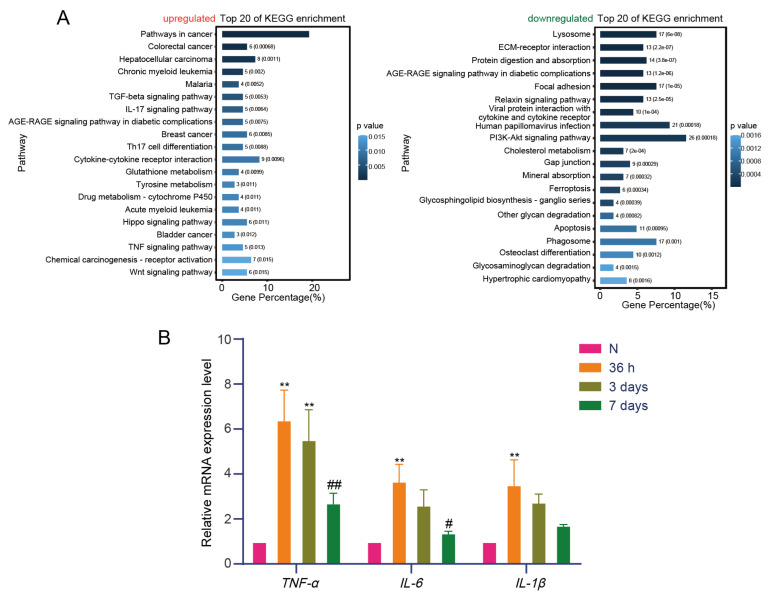
Inflammation is activated at an early stage. (A) Kyoto Encyclopedia of Genes and Genomes (KEGG) pathway analysis of upregulated and downregulated DEGs at 36 h. The top 20 KEGG pathways are displayed. (B) Inflammatory mRNA expression in atrophic skeletal muscle (n = 3 mice per group). **p < 0.01 versus the Ctrl group. # p < 0.05, ## p < 0.01 versus the 36 h group; one-way ANOVA with Tukey’s post hoc test.

Functional enrichment analysis was conducted on differentially expressed genes (DEGs) in the TA muscle at various time points following nerve injury. At 36 h, upregulated genes were primarily associated with inflammation-related pathways, including the TGF-β signaling pathway, which regulates inflammatory responses by modulating cytokine networks. TGF-β can attenuate acute inflammation by inhibiting the production of proinflammatory cytokines such as TNF-α, IL-1β, and IL-6 (Burks and Cohn, 2011). However, under chronic inflammatory conditions, TGF-β may promote tissue fibrosis, thereby exacerbating inflammation (Ismaeel et al., 2019). The IL-17 signaling pathway can directly stimulate the production of proinflammatory cytokines such as TNF-α, IL-1β, and IL-6, thereby exacerbating the inflammatory response (Toogood, 1989). IL-17 also promotes the expression of proinflammatory genes through activation of the NF-κB signaling pathway (Li et al., 2023). NF-κB is a key transcription factor that regulates the expression of numerous inflammation-related genes. In the TNF signaling pathway, TNF-α activates downstream signaling via TNF receptors (TNFR1 and TNFR2), leading to the release of proinflammatory factors (Collins and Grounds, 2001). TNF-α induces the secretion of cytokines such as IL-1, IL-6, and IL-8, thereby further amplifying the inflammatory response (Ji et al., 2022). Downregulated genes were enriched in the PI3K–Akt signaling pathway, a key regulator of muscle protein synthesis. These findings suggest that loss of innervation inhibits protein synthesis at early stages, thereby contributing to muscle atrophy (Vainshtein and Sandri, 2020; Sartori et al., 2021) (Figure 2A). Key inflammatory factors were validated, showing that proinflammatory cytokines such as TNF-α, IL-6, and IL-1β were upregulated at 36 h compared with the control group. Although their expression remained elevated at 3 and 7 days, all showed gradual downregulation as atrophy progressed. Early inflammation was activated, and the upregulation of several inflammatory factors was confirmed (Figure 2B).

### 3.3. Ankrd2 expression exhibits an opposite pattern to inflammatory factor expression

**Figure 3:**
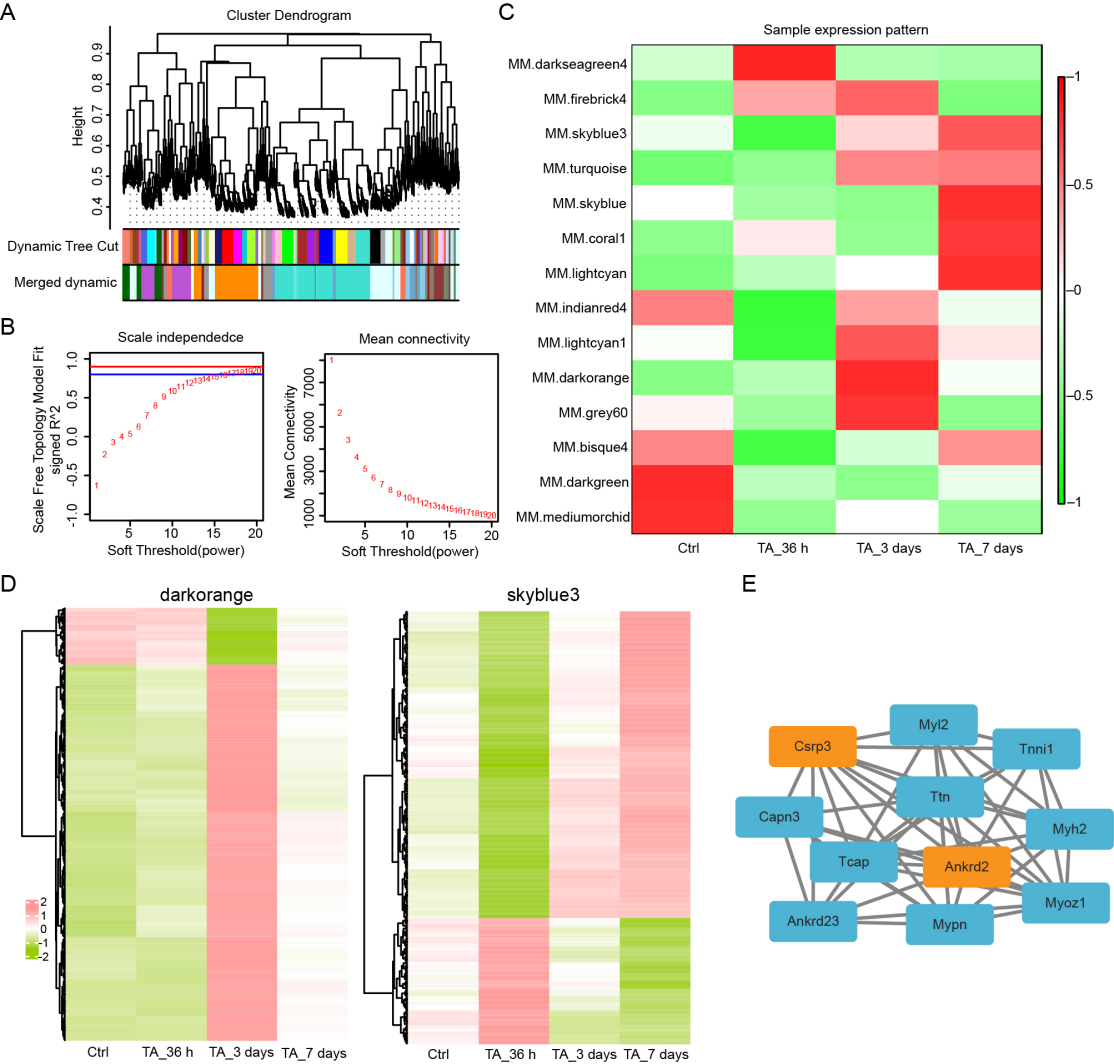
Weighted gene coexpression network analysis (WGCNA) of differentially expressed genes (DEGs). (A) DEG clustering dendrogram based on topological overlap dissimilarity. Dynamic tree cuts are applied to identify modules by dividing the dendrogram at important branch points. Modules are shown in different colors in the horizontal bar directly below the dendrogram. (B) Determination of the soft-thresholding power in WGCNA. The plot shows that a soft-thresholding power greater than 16 satisfies the scale-free topology criterion (R^2^ > 0.8). (C) Heatmap of correlations between samples and gene modules. Each row corresponds to a gene module, and each column corresponds to a sample. Each cell contains the corresponding correlation value. Red and green colors represent positive and negative correlations, respectively. The darker the color, the higher the correlation. (D) Heatmap showing expression patterns of DEGs at different time points in the darkorange and skyblue3 modules. (E) Proteins interacting with Ankrd2 were analyzed using the STRING database, with Ankrd2 and Csrp3 highlighted by orange boxes.

**Figure 4:**
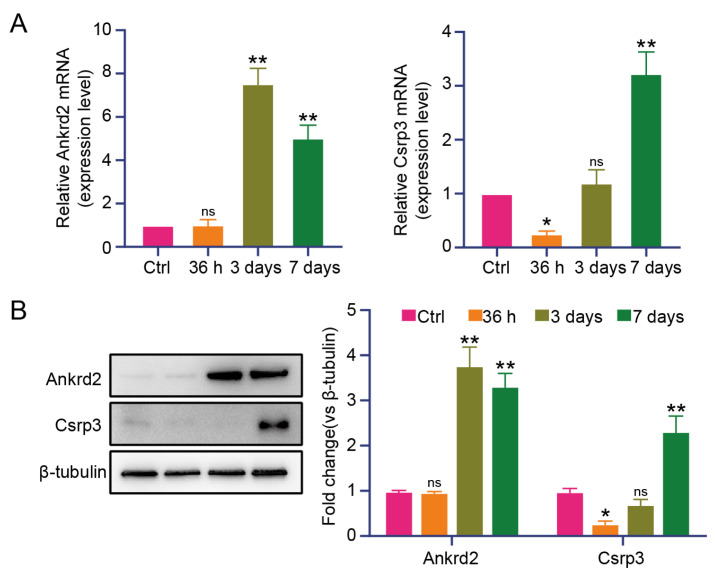
Expression of Ankrd2 and Csrp3 during skeletal muscle atrophy. (A) mRNA expression levels of Ankrd2 and Csrp3 during skeletal muscle atrophy, as determined by RT-qPCR. (B) Representative immunoblots and quantification of Ankrd2 and Csrp3 protein expression in the TA muscle at different time points. The control group represents the sham-operated group without nerve transection. Values are presented as mean ± SD; *p < 0.05, **p < 0.01 versus the control group; one-way ANOVA with Tukey’s post hoc test.

To identify key regulators of inflammatory expression, WGCNA was performed on differentially expressed genes (DEGs). WGCNA grouped the DEGs into 14 modules using a soft-threshold power of 16. Among these 14 modules, the darkorange and skyblue3 modules showed significant correlations with inflammatory factor expression and exhibited opposite expression patterns (Figures 3A–3C). Heatmap analysis of genes in these two modules revealed that the *Ankrd2* gene in the darkorange module and the *Csrp3* gene in the skyblue3 module may regulate inflammatory factor expression (Figure 3D). STRING database analysis revealed an interaction between Ankrd2 and Csrp3 (Figure 3E). Ankrd2 and Csrp3 expression in denervated TA muscle was further assessed using RT-qPCR and Western blot. Compared with the control group, Ankrd2 showed no significant change at 36 h but was upregulated at 3 and 7 days. Csrp3 displayed no significant change at 36 h or 3 days but was significantly upregulated at 7 days (Figures 4A and 4B). These findings indicate that Ankrd2 expression remained unchanged at early stages but increased at 3 and 7 days, whereas inflammatory factors exhibited a downregulation trend. These results suggest that upregulated Ankrd2 may contribute to the downregulation of inflammatory factors.

### 3.4. Overexpression of Ankrd2 alleviates denervation-induced skeletal muscle atrophy

**Figure 5:**
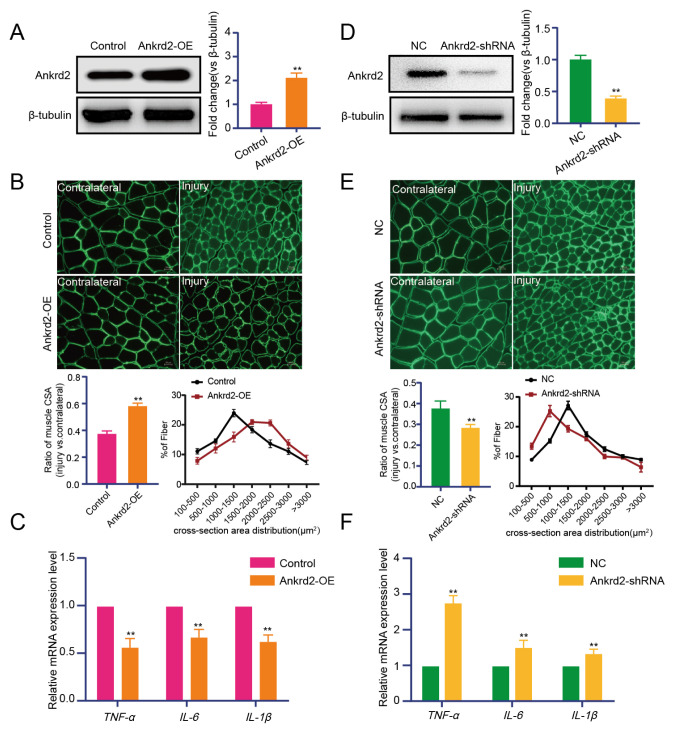
Overexpression of Ankrd2 alleviates denervation-induced skeletal muscle atrophy. (A/D) Ankrd2 protein expression in the TA muscles transfected with control lentivirus and Ankrd2 overexpression lentivirus, or Ankrd2-shRNA lentivirus and the corresponding negative control. (B/E) Immunofluorescence staining of laminin, cross-sectional area (CSA) measurements, and quantification of muscle fiber area distribution in skeletal muscle (n = 4 mice per group). Scale bar = 20 μm. (C/F) Inflammatory mRNA expression in the TA muscles of different experimental groups. Control: Denervated group injected with the corresponding negative control lentivirus (LV-NC). Ankrd2-OE: Denervated group injected with LV-Ankrd2. NC: Denervated group injected with the corresponding negative control lentivirus (shNC). shAnkrd2: Denervated group injected with shAnkrd2. Values are presented as mean ± SD; **p ≤ 0.01. versus the Ctrl or NC group; one-way ANOVA followed by Tukey’s post hoc test.

An Ankrd2-overexpressing lentivirus was injected into the TA muscle during establishment of the nerve disarticulation model to assess whether Ankrd2 overexpression protects against skeletal muscle atrophy. Tissue samples were collected 10 days after surgery for myofiber cross-sectional area analysis. Ankrd2 expression was first examined in the overexpression group. Western blot results showed that Ankrd2 protein levels were upregulated in the Ankrd2 overexpression group compared with the control group (Figure 5A). Analysis of muscle fiber cross-sectional area revealed that Ankrd2 overexpression increased muscle fiber size and attenuated skeletal muscle atrophy (Figure 5B). The effect of Ankrd2 overexpression on inflammatory factor expression was also examined. RT-qPCR analysis showed that Ankrd2 overexpression reduced the expression of TNF-α, IL-6, and IL-1β compared with the control group (Figure 5C). The effect of Ankrd2 inhibition on skeletal muscle atrophy was further examined. A nerve disarticulation model was established, and a lentivirus interfering with Ankrd2 expression was injected into the TA muscle, followed by similar analyses. Western blot results indicated that Ankrd2 protein expression was significantly reduced in the Ankrd2 interference group compared with the negative control group (Figure 5D). Analysis of muscle fiber cross-sectional area showed that Ankrd2 interference reduced muscle fiber size and exacerbated skeletal muscle atrophy (Figure 5E). Additionally, the expression levels of TNF-α, IL-6, and IL-1β were upregulated (Figure 5F).

### 3.5. Ankrd2 alleviates denervation-induced skeletal muscle atrophy potentially through downregulation of the NF-κB signaling pathway

**Figure 6:**
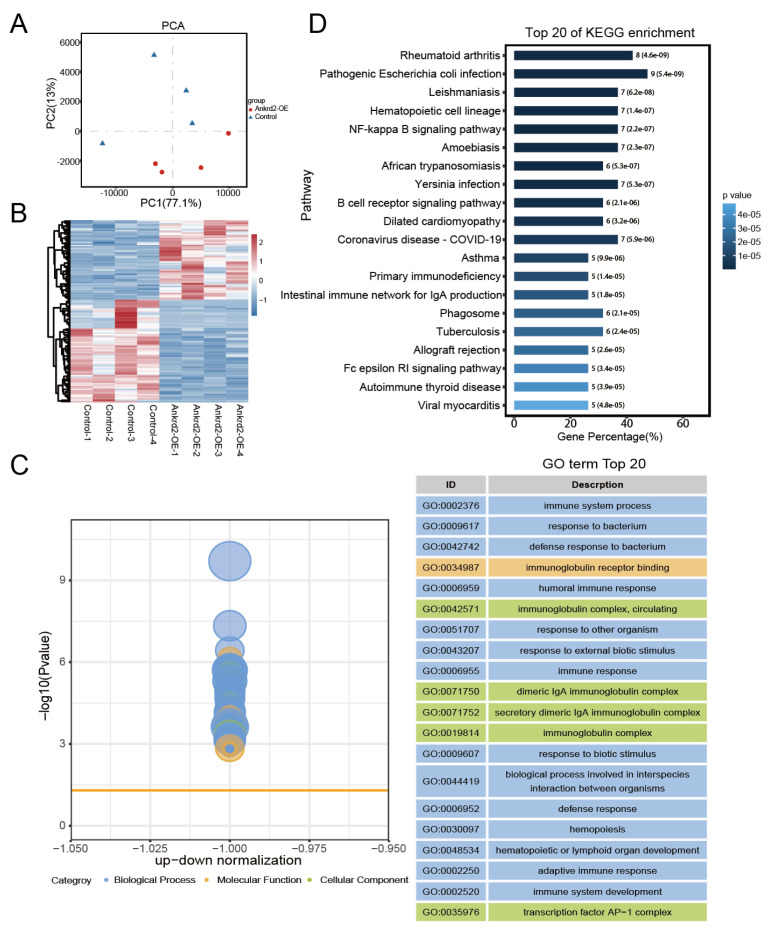
Functional analysis of differentially expressed genes in denervated skeletal muscle following Ankrd2 overexpression lentivirus treatment. (A) Principal component analysis (PCA) of RNA-sequencing samples. Shorter clustering distances indicate greater similarity between samples. (B) Heatmap of all differentially expressed genes between the control group and the Ankrd2 overexpression group. (C) Gene ontology (GO) enrichment analysis of differentially expressed genes in biological processes. (D) KEGG pathway enrichment analysis of downregulated genes.

**Figure 7:**
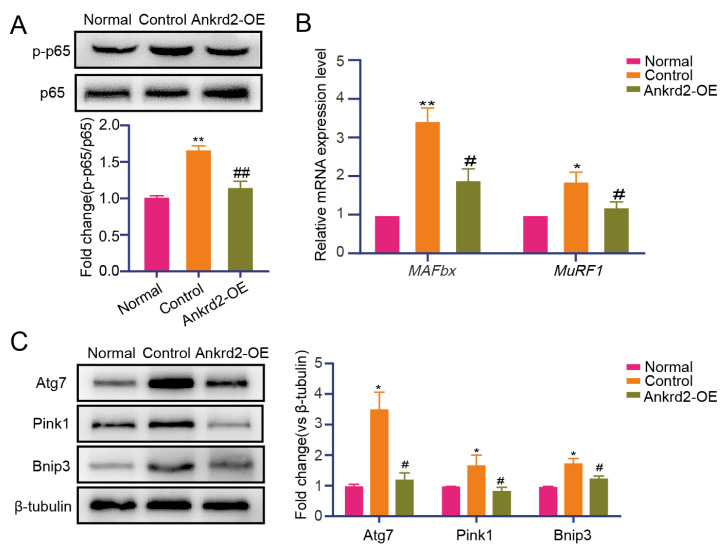
Ankrd2 downregulates the NF-κB signaling pathway to attenuate skeletal muscle atrophy. (A) Western blot analysis of p-p65 in the TA muscle of the normal, control, and Ankrd2 overexpression groups; total p65 was used for normalization. (B) RT-qPCR analysis of the E3 ubiquitin ligases MAFbx and MuRF1 in three experimental groups. (C) Western blot analysis of Atg7, Bnip3, and Pink1 protein levels in the TA muscle. β-tubulin was used as the loading control. Normal: Sham-operated, nondenervated group. Control: Denervated group injected with the corresponding negative control lentivirus (LV-NC). Ankrd2-OE: Denervated group injected with LV-Ankrd2. Values are presented as mean ± SD; *p ≤ 0.05, **p ≤ 0.01 versus the normal group; #p ≤ 0.05, ##p ≤ 0.01 versus the control group, as determined by unpaired student’s t-test.

To investigate the mechanism by which Ankrd2 alleviates skeletal muscle atrophy, transcriptome sequencing was performed in the Ankrd2 overexpression and control groups. Principal component analysis (PCA) showed clear separation between the Ankrd2 overexpression and control groups, indicating good intragroup consistency and significant intergroup differences (Figure 6A). Differential expression analysis identified 84 differentially expressed genes in the Ankrd2 overexpression group compared with the control group, including 50 upregulated and 34 downregulated genes, as illustrated by the heatmap (Figure 6B). GO analysis of downregulated genes revealed associations with multiple inflammation-related pathways. In BP analysis, enriched terms included immune system processes, humoral immune response, and immune response, whereas MF analysis highlighted immunoglobulin receptor binding, suggesting that Ankrd2 overexpression may reduce inflammatory receptor–mediated responses. CC analysis also linked downregulated genes to inflammation-related components, such as circulating immunoglobulin complexes (Figure 6C). KEGG analysis indicated that downregulated genes were primarily associated with the NF-κB signaling pathway (Figure 6D). Protein analysis showed that p-p65 expression was reduced in the Ankrd2 overexpression group compared with the control group (Figure 7A). Sciatic nerve injury triggered the secretion of inflammatory factors such as TNF-α, IL-6, and IL-1β, whereas Ankrd2 overexpression reduced inflammatory factor expression, inhibited activation of the NF-κB signaling pathway, decreased the expression of proatrophic genes in the ubiquitin–proteasome and autophagic–lysosomal systems, and ultimately alleviated skeletal muscle atrophy. As a transcription factor, NF-κB activates downstream proatrophy genes and regulates skeletal muscle atrophy under various pathological conditions, including denervation-induced atrophy. MAFbx and MuRF1, key E3 ubiquitin ligases in the ubiquitin–proteasome pathway that regulates protein degradation, were examined. Expression of both genes was reduced in the Ankrd2 overexpression group compared with the control group (Figure 7B). Proatrophy genes in the autophagic–lysosomal pathway, including Atg7, Bnip3, and Pink1, were also examined. Their expression was downregulated in the Ankrd2 overexpression group compared with the control group (Figure 7C). These findings suggest that Ankrd2 overexpression may alleviate denervation-induced skeletal muscle atrophy by reducing the inflammatory response and downregulating the NF-κB signaling pathway.

## 4. Discussion

**Figure 8:**
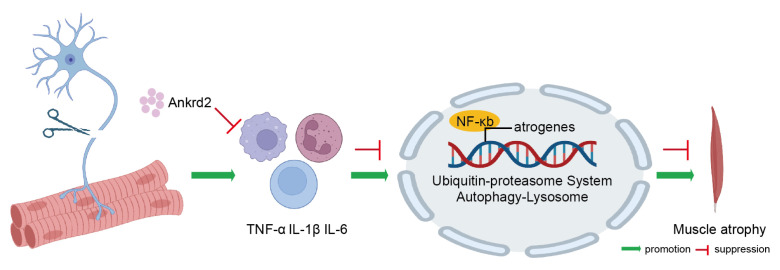
Proposed mechanism by which Ankrd2 alleviates denervation-induced skeletal muscle atrophy. Injury to the sciatic nerve triggers the secretion of inflammatory factors such as TNF-α, IL-6, and IL-1β. Overexpression of Ankrd2 protein reduces inflammatory factor expression, thereby inhibiting activation of the NF-κB signaling pathway, suppressing proatrophic gene expression in the ubiquitin–proteasome system and the autophagic–lysosomal system, and ultimately alleviating skeletal muscle atrophy.

When a nerve loses function, the affected muscle undergoes a series of changes that ultimately lead to atrophy. The effects of denervation extend beyond the muscle, affecting related physiological functions and motor abilities. Denervation may trigger an inflammatory response in muscle tissue, in which infiltrating cells release cytokines that impair muscle regeneration and may cause further damage and dysfunction (Rey-Garcia and Townsend, 2022). In some cases, antiinflammatory treatment may help to slow denervation-induced muscle atrophy and functional loss. However, the regulation of the inflammatory response remains incompletely understood, and further investigation into its regulatory mechanisms may offer new therapeutic strategies for denervated skeletal muscle atrophy. This study identified early-stage activation of inflammation, with several key inflammatory factors showing an upregulation pattern during atrophy. WGCNA screening identified Ankrd2 expression as being correlated with inflammatory factor expression patterns. Ankrd2 overexpression was shown to inhibit inflammatory factor expression and NF-κB signaling, as well as proatrophic genes involved in the regulation of skeletal muscle atrophy. These effects result in significant alleviation of denervation-induced skeletal muscle atrophy (Figure 8). Overall, this study suggests that Ankrd2 mitigates denervation-induced skeletal muscle atrophy by inhibiting inflammation, thereby enhancing understanding of its regulatory mechanisms and supporting its potential as a therapeutic target.

Sun et al. previously analyzed gene expression changes in skeletal muscle following denervation using gene microarrays, dividing the atrophy process into four stages and suggesting that the inflammatory response is activated early (Shen et al., 2019). A model of denervation-induced skeletal muscle atrophy was established, focusing on early and atrophic stages (36 h, 3 days, and 7 days), and transcriptome sequencing results were systematically analyzed. The results showed varying degrees of differential gene expression at all time points compared to the control group. Venn analysis of differentially expressed genes across groups revealed 242 commonly differentially expressed genes. Several upregulated genes (*Scn5a*, *Hdac4*, *Gadd45a*, and *Runx1*), previously reported as key regulators in denervation-induced skeletal muscle atrophy (Bongers et al., 2013), as well as downregulated genes (*Cdkn1c*, *Mbp*, *Mpz*, and *Nr4a1*), were selected for validation. Their expression levels were correlated with fragments per kilobase of exon per million mapped reads (FPKM) values obtained from sequencing data to verify the accuracy of the results. The results confirmed differential expression of the selected genes, as validated by RT-qPCR. Correlation analysis showed a positive correlation (R = 0.8869) between RT-qPCR validation results and FPKM values. These findings confirm the reliability of the sequencing data and support their use for further analyses.

The second phase following nerve loss involves activation of the inflammatory environment. During this phase, genes encoding inflammatory cytokines, such as tumor necrosis factor α (TNF-α) and transforming growth factor β (TGF-β), show increased expression (Shen et al., 2019). Muscle mass reduction is often linked to elevated proinflammatory cytokines, which are considered key mediators of muscle-specific proteolysis. For instance, elevated levels of proinflammatory cytokines (e.g., TNF-α, IL-1, and IL-6) in patients with cancer, diabetes, obesity, chronic kidney disease, or heart failure have been linked to reduced protein synthesis and enhanced protein degradation (Costamagna et al., 2015). In denervation-induced muscle atrophy, IL-6 alone can activate STAT3, which in turn triggers the ubiquitin–proteasome system and the autophagic–lysosomal system, both of which are involved in muscle atrophy (Wu et al., 2019). TNF-α, a well-studied proinflammatory cytokine, has significant effects on skeletal muscle. Elevated TNF-α typically induces muscle weakness through two mechanisms: promotion of atrophy and impairment of contractile function. In this study, skeletal muscle atrophy was primarily observed. Future studies may explore contractile function to further assess muscle performance using different indices. Additionally, TNF-α promotes protein loss at the cellular level through receptor-mediated signaling that alters muscle gene expression (Llovera et al., 1998). TNF-α plays a role in the pathology of various chronic diseases by inducing skeletal muscle protein degradation and reduced muscle mass, eventually leading to fatigue and impaired physical activity (Xiang et al., 2021). Pathological muscle damage triggers the release of cytokines that regulate the immune microenvironment in muscle tissue. A recent study identified increased RNA and protein levels of TNF-α in denervated muscle. To explore the role of TNF-α in denervation-induced muscle atrophy, C2C12 cells were treated with TNF-α during differentiation. It was observed that TNF-α significantly inhibited myotube formation in C2C12 cells in a dose-dependent manner, consistent with elevated TNF-α levels in denervated muscles (Li et al., 2023; You et al., 2023). Komiya et al. reported similar results, noting a significant increase in TNF-α levels 3 days postdenervation that persisted until day 14 (Komiya et al., 2019). IL-1β is a potent inducer of disease-related protein loss and muscle atrophy, with elevated IL-1β directly contributing to muscle atrophy. Additionally, IL-1β induces the production of immune factors such as TNF-α, IFN-γ, IL-6, IL-4, and nerve growth factor and activates cyclooxygenase-2 (COX-2), thereby exacerbating or mitigating nerve damage (Aneas et al., 2021). You et al. observed, in a model of neurite loss–induced muscular dystrophy, that IL-1β protein and mRNA levels gradually increased and peaked at 28 days postneuronal loss (You et al., 2023). In this study, rapid upregulation of TNF-α, IL-1β, and IL-6 was observed at 36 h postdenervation, followed by varying degrees of downregulation at 3 and 7 days compared with 36 h. These findings suggest the involvement of key regulatory factors in modulating the altered expression of inflammatory cytokines.

To identify key factors that may regulate the inflammatory response, WGCNA was performed. In total, 14 expression modules were identified, among which the darkorange and skyblue3 modules showed significant correlations with inflammatory factor expression patterns. After screening genes within these two modules and performing heatmap analysis, Ankrd2 in the darkorange module and Csrp3 in the skyblue3 module were identified as potential regulators of inflammatory factor expression. STRING database analysis revealed an interaction between Ankrd2 and Csrp3. Ankrd2 is a key structural protein in skeletal muscle and plays a crucial role in muscle fiber integrity. It is part of a stretch-sensing complex linked to titin, enabling muscles to detect and transmit mechanical signals during stretch. This structural interaction supports the mechanical strength of skeletal muscle and promotes muscle function and growth (Jasnic-Savovic et al., 2015). Ankrd2 also acts as a nuclear cotranscription factor, regulating gene expression related to muscle development. During muscle differentiation, Ankrd2 promotes specific gene expression by interacting with transcription factors, thereby driving muscle cell proliferation and differentiation. This process is crucial for normal muscle development (Cenni et al., 2019; Mohamed et al., 2010). Ankrd2 function extends beyond normal muscle development and plays a significant role in various disease states. In muscle diseases such as atrophy or injury, Ankrd2 expression often increases, indicating an adaptive response to pathological conditions (Mohamed et al., 2013). In this study, Ankrd2 expression showed no significant change at 36 h compared with the control group but gradually increased at 3 and 7 days. Ankrd2 overexpression significantly mitigated skeletal muscle atrophy and reduced inflammatory factor expression. These results suggest that Ankrd2 tends to be upregulated during denervation-induced skeletal muscle atrophy and that its overexpression exerts a protective effect. Other factors, such as FGF21, are also upregulated during skeletal muscle damage and exhibit protective roles. Although FGF21 is expressed at very low levels under basal conditions, it appears to contribute to muscle homeostasis when induced by muscle stress (Fisher and Maratos-Flier, 2016). FGF21 regulates multiple signaling pathways linked to muscle metabolism, including the AMPK and mTOR pathways. These pathways are critical for cellular energy homeostasis and growth regulation. Through regulation of these pathways, FGF21 aids muscle cell survival in adverse environments following nerve injury (Pereira et al., 2017). The sequencing results indicated a moderate upregulation of FGF21 in skeletal muscle following loss of innervation. CSRP3 has been identified as a key regulator in the physiology and pathophysiology of rhabdomyosin (Flick and Konieczny, 2000). Mutations in Csrp3 can directly cause human cardiac and skeletal muscle diseases (Arber et al., 1997). Evidence also suggests that interactions between Csrp3 and MyoD are crucial for myogenic differentiation and muscle cell remodeling (Kong et al., 1997). Silencing of Csrp3 results in downregulation of myogenic gene expression and upregulation of atrophy-related genes. Csrp3 has been shown to interact with LC3 proteins, thereby promoting autophagosome formation during autophagy. Csrp3 silencing impairs autophagy in myofibroblasts, as evidenced by suppression of autophagy-related Atg5 and Atg7 mRNA expression and inhibition of LC3-II and Beclin-1 protein accumulation (Cui et al., 2020). In this study, Csrp3 exhibited low expression at 36 h and 3 days, with upregulation observed at 7 days, contrasting with the expression patterns of inflammatory factors. An interaction between Ankrd2 and Csrp3 was also identified. Given the interaction between Ankrd2 and Csrp3, it is plausible that they may function as a coregulatory complex in response to denervation stress, potentially comodulating inflammatory signaling and muscle integrity. This hypothesis, which integrates the observed expression patterns and their physical interaction, warrants further investigation into their combined role in atrophy regulation.

Ankrd2 is a member of the muscle ankyrin repeat protein (MARP) family and participates in muscle stress response pathways, coordinating myocyte proliferation and apoptosis during myogenic differentiation. Ankrd2 has been shown to negatively regulate the expression of numerous genes involved in NF-κB-mediated inflammatory pathways. NF-κB influences skeletal muscle atrophy by regulating the expression of various proatrophic genes. Inhibition of the NF-κB signaling pathway effectively alleviates skeletal muscle atrophy (Thoma and Lightfoot, 2018; Fang et al., 2021). While the findings regarding Ankrd2’s protective role align with its previously established functions in mechanotransduction and muscle differentiation, they also reveal a novel dimension of its activity. Prior research has primarily focused on the role of Ankrd2 in development, responses to mechanical stress, or muscular dystrophies. This study demonstrates that Ankrd2 is upregulated in a time-dependent manner in denervated muscle and that its overexpression can counteract atrophy by suppressing inflammation. This positions Ankrd2 not merely as a marker of muscle pathology but as a functional modulator of the inflammatory response in the context of denervation, a mechanism that has remained largely unexplored. In this study, transcriptome sequencing analysis was conducted on TA muscle injected with an Ankrd2-overexpressing lentivirus to investigate pathways altered by this treatment and the mechanisms involved in alleviating denervation-induced skeletal muscle atrophy. Downregulated genes were subjected to KEGG functional analysis, revealing inhibition of the NF-κB signaling pathway. An imbalance between protein synthesis and degradation pathways in muscle tissue is a significant contributor to skeletal muscle atrophy. Denervation significantly increases the expression of muscle-specific atrophy genes MAFbx and MuRF1. Genetic deletion of MAFbx and MuRF1 has been shown to alleviate denervation-induced muscle atrophy in mice (Bodine and Baehr, 2014). These proatrophy genes are regulated by the transcription factor NF-κB. Based on the sequencing results, the expression of p-p65, MAFbx, and MuRF1 was examined. The findings indicated that Ankrd2 overexpression reduced the levels of p-p65, MAFbx, and MuRF1 compared with the negative control group. The autophagic–lysosomal system is another key pathway regulating skeletal muscle atrophy. Several major proatrophy genes within this system were examined, and the expression of Atg7, Bnip3, and Pink1 was found to be suppressed compared with the negative control group. In this study, the ultrastructure of mitochondria related to autophagy in skeletal muscle was not examined in depth, representing an important direction for future investigations.

Despite the compelling evidence presented here, this study has limitations that point to important directions for future research. The most significant gap concerns the precise molecular mechanism by which Ankrd2 influences the NF-κB pathway. The data strongly support a functional link but do not delineate the direct protein interactions involved. Future studies should employ coimmunoprecipitation coupled with mass spectrometry to identify direct binding partners of Ankrd2 in atrophying muscle. Furthermore, luciferase reporter assays would be invaluable to confirm the direct inhibitory effect of Ankrd2 on NF-κB transcriptional activity. Investigating whether Ankrd2 affects the phosphorylation, ubiquitination, or degradation of IκBα will also be critical to map its exact position within the NF-κB signaling cascade. Addressing these questions will be essential to fully realize the therapeutic potential of targeting Ankrd2.

## 5. Conclusions

This study provides evidence of a protective effect of Ankrd2 against denervated skeletal muscle atrophy. Although Ankrd2 expression is upregulated in the late stages of atrophy, its overexpression can alleviate skeletal muscle atrophy and reduce inflammatory factor expression. This reduction subsequently decreases the expression of proatrophic genes within the NF-κB signaling pathway, as illustrated in the schematic diagram (Figure 8). Ankrd2 has varying effects on skeletal muscle across different physiological states. This study indicates that Ankrd2 may alleviate inflammatory factor expression. A deeper understanding of the molecular regulatory mechanisms of Ankrd2 will be essential for the development of therapeutic strategies for muscle diseases.

## Figures and Tables

**Table:** Primer sequences used for RT-qPCR.

Gene	Sequence 5′-3′(Forward)	Sequence 5′-3′ (Reverse)
*Scn5a*	ATGACAGCCGAGTTTGAGGA	GCTGAGGATGACGATGATGC
*Hdac4*	TGTGGTACTGGTGTCATCGG	TCAGAAGCATCACAGATGGC
*Gadd45a*	TGCTACTGGAGAACGACGC	CCCACTGATCCATGTAGCGA
*Runx1*	GACAGCCCCAACTTCCTCT	GCTACCTGGTTCTTCATGGC
*Cdkn1c*	CGAGGAGCAGGACGAGAATC	CTTGTTCTCCTGCGCAGTTC
*Mbp*	TCACACACGAGAACTACCCA	CTTGGGATGGAGGTGGTGTT
*Mpz*	AGACTACAGTGACAACGGCA	AGAAGAGCAACAGCAGCAAC
*Ankrd2*	CTGCAGTGGAGGGGAAAATG	ATCTCCATGTGTCCCTCCAG
*Csrp3*	GTGGAGGTGCAAAATGTGGA	TCTCTGACTCATGAGCTGCC
*MAFbx*	TGTGGGTGTATCGGATGGAG	ACAGGCAGGTCGGTGATC
*MuRF1*	AAGTGATCATGGACCGGCA	CGTCTTCGTGTTCCTTGCAC
*Gapdh*	CATCACTGCCACCCAGAAGACTG	ATGCCAGTGAGCTTCCCGTTCAG
